# Chlorophyll fluorescence emission can screen cold tolerance of cold acclimated *Arabidopsis thaliana* accessions

**DOI:** 10.1186/1746-4811-10-38

**Published:** 2014-11-06

**Authors:** Anamika Mishra, Arnd G Heyer, Kumud B Mishra

**Affiliations:** Global Change Research Centre, Academy of Sciences of the Czech Republic, v. v. i, Bělidla 986/4a, 603 00 Brno, Czech Republic; Institute of Biomaterials and Biomolecular Systems, Department of Plant Biotechnology, University of Stuttgart, Stuttgart, Germany

**Keywords:** High-throughput screening, Chlorophyll *a* fluorescence transients, Cold tolerance, Cold acclimation, Whole plant, *Arabidopsis thaliana*

## Abstract

**Background:**

An easy and non-invasive method for measuring plant cold tolerance is highly valuable to instigate research targeting breeding of cold tolerant crops. Traditional methods are labor intensive, time-consuming and thereby of limited value for large scale screening. Here, we have tested the capacity of chlorophyll *a* fluorescence (ChlF) imaging based methods for the first time on intact whole plants and employed advanced statistical classifiers and feature selection rules for finding combinations of images able to discriminate cold tolerant and cold sensitive plants.

**Results:**

ChlF emission from intact whole plant rosettes of nine *Arabidopsis thaliana* accessions was measured for (1) non-acclimated (NAC, six week old plants grown at room temperature), (2) cold acclimated (AC, NAC plants acclimated at 4°C for two weeks), and (3) sub-zero temperature (ST) treated (STT, AC plants treated at -4°C for 8 h in dark) states. Cold acclimation broadened the slow phase of ChlF transients in cold sensitive (Co, C24, Can and Cvi) *A. thaliana* accessions. Similar broadening in the slow phase of ChlF transients was observed in cold tolerant (Col, Rsch, and Te) plants following ST treatments. ChlF parameters: *maximum quantum yield of PSII photochemistry* (F_V_/F_M_) and *fluorescence decrease ratio* (R_FD_) well categorized the cold sensitive and tolerant plants when measured in STT state. We trained a range of statistical classifiers with the sequence of captured ChlF images and selected a high performing *quadratic discriminant classifier* (QDC) in combination with *sequential forward floating selection* (SFFS) feature selection methods and found that linear combination of three images showed a reasonable contrast between cold sensitive and tolerant *A. thaliana* accessions for AC as well as for STT states.

**Conclusions:**

ChlF transients measured for an intact whole plant is important for understanding the impact of cold acclimation on photosynthetic processes. Combinatorial imaging combined with statistical classifiers and feature selection methods worked well for the screening of cold tolerance without exposing plants to sub-zero temperatures. This opens up new possibilities for high-throughput monitoring of whole plants cold tolerance *via* easy and fully non-invasive means.

## Background

Cold tolerance is the ability of plants to withstand low temperatures and plays a crucial role in worldwide production of many important agricultural crops. In temperate climate zones, plants have developed strategies to adjust low temperature tolerance by employing a highly complex process of physiological re-arrangements, termed cold acclimation that is triggered by low but non-freezing temperatures. Numerous studies revealed that cold acclimation not only programs massive changes in transcriptome and metabolome [1–7] but also induces structural and compositional modifications of compatible solutes in various sub-cellular compartments [8]. Following acclimation, plants are more efficient in dealing with the impact of sustained cold or sudden temperature drops by activating a plethora of adjustments including accumulation of cryoprotective metabolites and proteins for their survival [9]. A correlation of cold acclimation capacities with habitat winter temperatures points to high metabolic or ecological costs of these adjustments [5, 10, 11]. Despite intensive research the molecular mechanisms of cold tolerance are still not fully understood and remain an area for intensive research, as understanding of mechanisms responsible for cold acclimation would allow breeding of cold tolerant crops.

The primary requirement for research associated with engineering of low temperature tolerant crops is to develop efficient and cost effective methods for measuring cold tolerance. Measurement of survival or re-growth scores following triggers of cold acclimation and de-acclimation treatments are quite lengthy and may not be very accurate [12, 13]. Most quantitative methods for assessing cold tolerance employ analysis of damage to plasma membranes by electrolyte leakage (EL) screens or the thylakoids *via* plastocyanin release after freeze-thaw cycles [14]. Generally, the temperature affording half of maximal damage (LT_50_), i.e. the temperature at which 50% of electrolytes or plastocyanin are released from their respective compartments, is being evaluated and used as a proxy for cold tolerance. However, since the measurement of purified plastocyanin is comparatively difficult [15], the most popular method to study plant cold tolerance is the evaluation of EL of detached leaves by using conductivity measurements [5, 16]. According to this method, detached leaves are placed in reaction tubes, which are progressively cooled down to certain temperatures. Low-temperature treated samples are then thawed at 4°C and electrical conductivity of a bathing solution is measured to calculate LT_50_ values that represent half-maximum damage to the plasma membrane [16]. EL is a widely accepted method for quantification of plant cold tolerance, although its results sometimes deviate from those obtained with alternative methods [17, 18].

The response of thylakoids membranes to sustained low-temperature treatments can be measured non-invasively by the analysis of ChlF emission. ChlF is re-emitted by chlorophyll *a* molecules following light absorption, and it is modulated by photochemical and non - photochemical events in the photosynthetic pigment–protein complexes PSII and PSI of the thylakoid membranes [19, 20]. The relative contribution of PSII and PSI in ChlF emission is also reported to change during cooling treatments [21]; thereby, this method can provide important insights into molecular processes of cold acclimation. The ChlF parameter F_V_/F_M_ of detached leaves following freeze - thaw cycles has successfully been applied to quantify cold tolerance in *Arabidopsis thaliana* as well as in other plant species [reviewed in [8, 22–24]. The analysis of polyphasic fluorescence rise of from initial low fluorescence (F_O_) to peak F_P_ by JIP test [25] has also been reported to be useful for the selection of cold tolerance in wheat genotypes [26, 27]. Earlier, we measured the ChlF transients of detached leaves of differentially cold tolerant *A. thaliana* accessions during progressive cooling starting from room temperature to -15°C and found high correlation between LT_50_ measured by electrolyte leakage with ChlF parameters such as F_O_(-15°C)/F_O_(4°C) and [F_S_/F_O_]_-15°C_ [10]. We demonstrated that application of advanced statistics-based combinatorial imaging methods to the sequence of time resolved ChlF images could be used to categorize cold tolerance levels by training of classifiers using fluorescence emission of detached leaves that were slowly cooled at mild sub-zero-temperatures of around -4°C [10]. Vaclavik et al. [7] reported that cold acclimation induced accumulation of Gluconapin and Flavon-3-ol glycosides, respectively, in cold tolerant vs. cold sensitive *A. thaliana* accessions, and thus metabolomic patterns, could be used for the screening of cold tolerance already in the cold acclimated state i.e. without exposing plants to freezing temperatures. Although metabolomic based methods can thus have better discriminating capacity of cold tolerant and cold sensitive accessions as compared to EL methods, they are not readily applicable in high-throughput screenings. Therefore, in the search for easy methods of sensing cold tolerance, we have tested the potential of ChlF transients measured on whole plant rosettes of the model species *Arabidopsis thaliana*. We involved NAC, AC, and STT plants of nine *A. thaliana* accessions that span the north–south range of the species [5]. A short light/dark protocol was applied for recording of ChlF transients of dark-adapted plants for about 202 s using a pulse amplitude modulation (PAM) based fluorometer [28]. We found that ChlF transients averaged over whole intact plant rosettes offered valuable information for assessing impacts of cold acclimation and cold induced alterations of the photosynthetic machinery. In addition to classical analysis of ChlF transients, we employed statistical classifiers and feature selection methods on captured ChlF images to search for highly contrasting features of cold tolerant vs. cold sensitive accessions. We have trained several classifiers and chose the best performing classifier, i.e. a QDC, for further use along with a high performing SFFS feature selection methods to identify features correlated with cold tolerance. The sets of images obtained for the STT state were then tested on AC state data sets, and we found that this method, i.e. combinatorial imaging, can be applied for assessing plant cold tolerance already in the cold acclimated state, i.e. without any sub-zero temperature treatments and by fully non-invasive means.

## Results

### ChlF transients from whole plants are highly informative for categorization of cold tolerance in *A. thaliana*accessions

**Figure 1:**
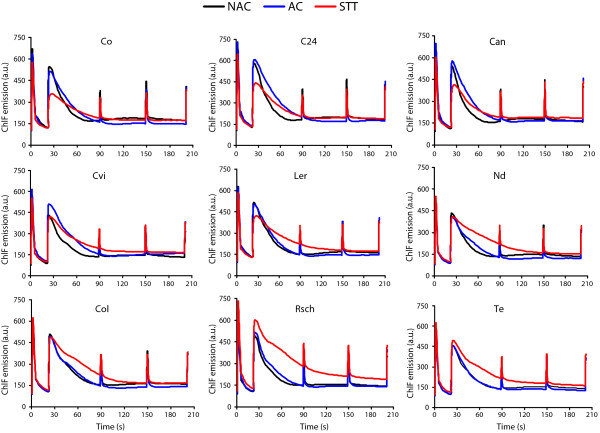
**The chlorophyll fluorescence (ChlF) transients of nine differentially cold tolerant** ***Arabidopsis thaliana*** **accessions measured by short experimental light/dark protocol.** The cold sensitive accessions are Co, C24, Can and Cvi (d); intermediate cold tolerant accession are Ler and Nd; and cold tolerant accessions are Col, Rsch and Te. The ChlF transient was integrated across whole plant rosettes and mean of three independent plants for each accession was presented.

We measured the ChlF transients of whole plant rosettes in nine differentially cold tolerant *Arabidopsis thaliana* accessions in the NAC, AC, and STT state [Figure 1]. From the shape of ChlF transients it can be established that for the cold sensitive accessions (Co, C24, Can, and Cvi), the fluorescence intensity following peak F_P_ quenched slowly in cold acclimated (AC) state, whereas the value of F_P_ itself decreased significantly when these plants were treated with sub-zero temperature (ST, -4°C for 8 h in dark). In cold tolerant accessions (Te, Rsch, and Col-0), however, the shape of the ChlF transients remained similar for NAC and AC states; while after STT, quenching of fluorescence intensity following peak F_P_ is significantly slowed down. Qualitative comparison of the ChlF transients of intermediate accessions (Ler and Nd) revealed a compound picture, and based on qualitative comparisons we tend to assign Ler as cold sensitive, while Nd can be classified cold tolerant.

### ChlF parameters F_V_/F_M_ and R_FD_ of whole plant rosettes can measure cold tolerance in subzero-temperature (ST) treated plants

**Figure 2:**
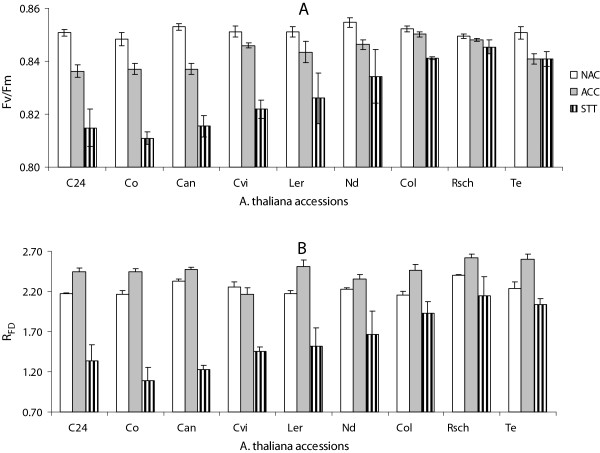
**Chlorophyll fluorescence (ChlF) parameters** ***: maximum quantum yield of PSII photosystems*** **(F**_**V**_ **/F**_**M**_ **) [A] and** ***fluorescence decrease ratio*** **(R**_**FD**_ **) [B] of differentially cold tolerant accessions of** ***Arabidopsis thaliana*** **for non-acclimated (NAC), cold acclimated (AC), and sub-zero temperature treated (STT) states.** The presented numeric values are mean of three independent plants with standard errors, and are integrated across whole plant rosettes.

Fluorescence parameters F_V_/F_M_ and R_FD_ were evaluated from measured ChlF emission and presented as an average of three independent experiments, where values of each parameter were estimated by integrating across whole rosette leaves. We did not find correlations between the classical ChlF parameters and cold tolerance among the investigated *A. thaliana* accessions either for NAC or for the AC state. However, the ChlF parameters F_V_/F_M_ and R_FD_, differed for cold sensitive, intermediate and cold tolerant accessions following STT (Figure 2). In STT state, F_V_/F_M_ values for cold tolerant (Te, Col and Rsch), intermediate (Nd and Ler) and sensitive accessions (Co, C24, and Can) were around 0.85 ± 0.01, 0.83 ± 0.01 and 0.81 ± 0.01, respectively, while the averaged R_FD_ values were calculated as 1.98 ± 0.19, 1.48 ± 0.07 and 1.40 ± 0.19, respectively. Non-paired t-test revealed F_V_/F_M_ to differ significantly (p <0.01) for cold tolerant and cold sensitive accessions, while there was no significant difference between cold sensitive and intermediate or intermediate and tolerant accessions. In contrast, plant vitality index R_FD_ showed significant differences between the cold sensitive and tolerant as well as between intermediate and tolerant accessions (p <0.05), but no significant difference was found between sensitive and intermediate tolerant accessions. For the accessions Cvi the values of ChlF parameters F_V_/F_M_ and R_FD_ are almost similar to that of intermediate accessions Ler and Nd, thereby its tolerance level of thylakoid may be categorized intermediate, while the plasma membrane behaved cold sensitive in EL measurements.

### Cold acclimation induced gain in photosynthetic performance of *A. thaliana*accessions

**Figure 3:**
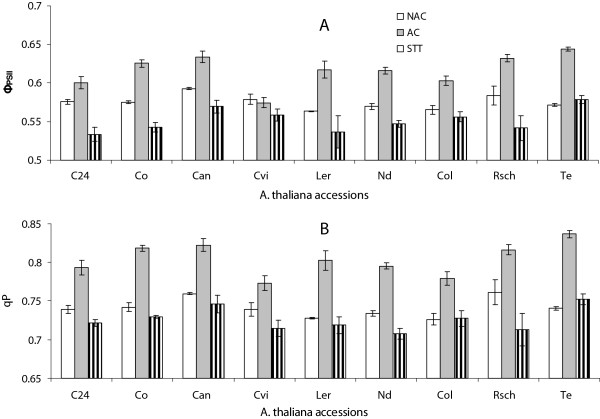
**The** ***effective quantum efficiency PS II*** **(Φ**_**PSII**_ **) [A] and** ***photochemical quenching*** **(qP) [B] of cold sensitive (Co, C24, Cvi, Can), intermediate cold tolerant (Nd and Ler) and cold tolerant (Te, Rsch and Col)** ***Arabidopsis thaliana*** **accessions for non-acclimated (NAC), cold acclimated (ACC), and sub-zero temperature treated (STT) states.** The presented numeric values are integrated across whole plant rosettes and mean of three independent plants with standard errors.

The ChlF parameter *effective quantum efficiency of PS II photochemistry* (Ф_PSII_) was significantly higher (p <0.01) in AC vs. NAC plants except for Cvi (Figure 3A). Interestingly, mild sub-zero temperature treatments (-4°C) for eight hours in AC plants led to a significant decline in the value of Ф_PSII_ in all accessions (Figure 3A) without Ф_PSII_ being correlated with the cold tolerance. Two weeks of cold acclimation caused a significant (p <0.01) increase of *photochemical quenching* (qP) in all accessions, while STT led to a decline (Figure 3B). As for Ф_PSII_, no correlation of parameter qP with cold tolerance was observed.

### Combinatorial imaging

**Figure 4:**
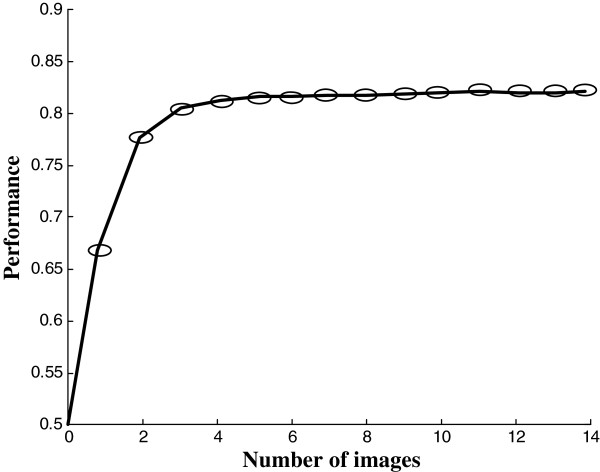
**The performance curve of** ***sequential forward floating selection*** **(SFFS) with images in the x-axis.**

**Figure 5:**
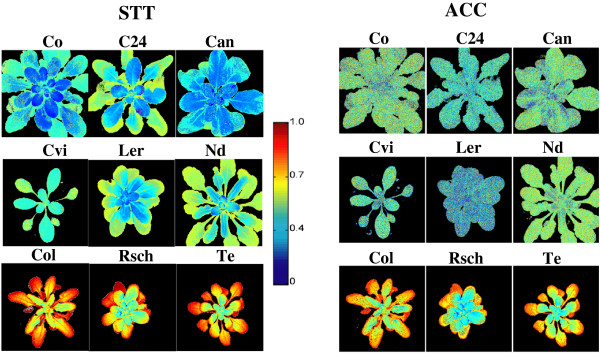
**Illustrates the combination of three most contrasting images for all nine accessions of** ***Arabidopsis thaliana*** **.** After subzero temperature treatment the all nine accession has been visibly divided in three categories namely, most sensitive (Co, C24 and Can), intermediates (Ler and Nd) and the most tolerant accessions (Te, Rsch and Col).

We applied statistical techniques of classifier and feature selection methods in order to identify features of cold tolerance from captured sequences of time-resolved ChlF images. This method is very powerful in identifying image sets from large sequences of time-resolved ChlF recordings that yield the highest contrast between groups to be compared. Earlier we demonstrated the usefulness of this approach for discriminating three species of family *Lamiaceae* at very early stages of growth [29] and for investigating features of cold tolerance at non-lethal temperatures [10]. The method depends on training and performance testing of randomly selected pixels of the most contrasting ChlF image sets, i.e. recordings for the highly cold tolerant accession Te and highly sensitive Co in the STT state [for technical details, see [10, 29, 30]. The performance, error rate and computational time to run the algorithms of five tested classifiers, *linear discriminant classifier* (LDC), *quadratic discriminant classifier* (QDC), *k*-*nearest neighbors classifier* (*k*-NNC), *nearest neighbor classifier* (NNC) and *nearest mean classifier* (NMC), are presented in Table 1. We found that algorithms of QDC are the best performing classifier (81% correct assignment of cold tolerance group among test images) in comparatively short time (<6 s, Table 1). Therefore, QDC was chosen and applied with the SFFS feature selection method to find the most contrasting image sets for highly cold tolerant (Te) vs. highly cold sensitive (Co) accessions. Figure 4 shows the performance curve for the most efficient feature selection method SFFS in combination with simulated classifier QDC, where x-axis represents number of images. Thus, in this experiment, the SFFS algorithm reduced the full data set of 218 images to three images identified as I_21,_ I_104_ and I_107_ without compromising the classification performance (~80%, Figure 4). We obtained the linear combination: C = (0.3219)*I_21_ + (-0.5018)*I_104_+ (-0.2315)*I_107_, to perform best in discriminating cold tolerant and sensitive accessions. The coefficients of the linear combination were calculated according to Matouš et al. [30]. Figure 5 shows linear combinations of images for all nine accessions in the STT as well as AC state. The presented images are plotted on a false color scale where color represents the virtual fluorescence intensity of the pixel with reference to its corresponding value in the training data set. Thus, we can visualize a clear difference between cold sensitive, intermediate, and cold tolerant accessions in the STT state. When the combinatorial imaging was applied to AC plants it apparently failed to discriminate cold sensitive and intermediate accessions showing very similar patterns for leaf rosettes of the two groups of plants. However, the tolerant group of accessions could be clearly discriminated already in the AC state.

**Table 1:** **The table presents performance, error rate and computational time of the tested classifiers**

Classifiers	Performance	Error rate	Computational time (s)
LDC	0.81	0.09	6.14
QDC	0.81	0.09	5.86
*k*-NNC (*k* = 3)	0.82	0.08	5530
NNC (k = 1)	0.79	0.10	5510
NMC	0.47	0.26	8.23

## Discussion

### Towards easy methods for assessing whole plant cold tolerance in natural accessions of *A. thaliana*

In an attempt to establish an easy and non-invasive method for screening plant cold tolerance, we compared different concepts for measuring cold tolerance based on chlorophyll fluorescence that have been approved for detached leaves of the model species *Arabidopsis thaliana*. As far as we know, for the first time we attempted to capture the ChlF transients of intact whole plant rosettes and found interesting modulation in it for the group of cold sensitive (Co, C24, Can, Cvi), intermediate tolerant (Ler, Nd) and cold tolerant (Te, Rsch, Col-0) *A. thaliana* accessions for NAC, AC and STT state (Figure 1). The ChlF transient measured by quenching analysis of modulated fluorescence is very useful methods that can monitor slight changes in the photochemical and non-photochemical process [31]. The slow quenching of ChlF transients following peak F_P_, i.e. broadening of ChlF transients, for cold sensitive group of accessions under cold acclimated state and for tolerant group of accessions under STT state (Figure 1) is complex and can be affected by series of events such as light induced intrathylakoid H^+^ concentration, non-photochemical quenching, inhibition of CO_2_ assimilation processes, ATP synthesis among others [reviewed in [32]. However, it reveals that sensitive and tolerant group of accessions possess two different strategies to utilize the absorbed irradiance following cold acclimation as well as when placed at mild subzero temperature (-4°C) for 8 h in darkness. Probably cold tolerant plants efficiently utilize acclimation temperature and keeping homeostasis of photosynthetic apparatus and yielded almost similar ChlF transients in NAC as well as in AC state that varied only when treated with sub-zero temperature. The reduction of F_P_ for the group of cold sensitive accessions in STT state indicates perturbation in PSII functional properties. Significant inhibition of F_V_/F_M_ for sensitive vs. tolerant accessions in STT states further support slight inactivation of PSII function. Ehlert and Hincha [23] reported that the easily recordable parameter F_V_/F_M_ discriminates cold tolerant and sensitive detached leaves after a freeze-thaw cycle. Using a set of nine differentially cold tolerant natural accessions of *Arabidopsis* [5], we could verify also for whole plants that F_V_/F_M_ differs for tolerant and sensitive accessions after a sub-zero temperature treatment, but plants with intermediate tolerance differed neither from sensitive nor from tolerant ones. It has been reported that the fluorescence decrease ratio, R_FD_, is more sensitive to a variety of stress factors than F_V_/F_M_, which mainly responds to extreme conditions [33, 34]. Indeed, R_FD_, which can be calculated from fluorescence peak F_P_ and F_S_ measured under prevailing actinic light, was not only able to discriminate sensitive and tolerant but also tolerant and intermediate accessions. Interestingly, both F_V_/F_M_ and R_FD_ data revealed that plastids of the accession Cvi behaved as intermediately tolerant, while the electrolyte leakage method classified this accession as sensitive based on damage to the plasma membrane during freeze-thaw treatments [5]. We have earlier reported that cold tolerance of plastids and the plasma membrane appear not strictly correlated in the accession Col-0 [8], and the differential tolerance of plastids and plasma membrane in Cvi is further evidence for independent principles underlying cold tolerance of the different types of membranes.

Considering plants not treated with sub-zero temperatures, which would be desirable for a cold tolerance screening program, neither F_V_/F_M_ nor R_FD_ were correlated with tolerance of the nine *Arabidopsis* accessions used in this study. Since both parameters are related to intactness of PSII, it is not unexpected that they are not affected by above-zero temperatures in a cold tolerant plant like *Arabidopsis*. However, it was noticed that parameters Ф_PSII_, and qP that reflect effects of actinic light on chlorophyll fluorescence responded differentially to low above-zero and sub-zero temperature treatments. It has been reported that shifting plants from normal to low temperatures causes repression of genes related to photosynthesis [35], and among them are genes directly involved in light harvesting like e.g. proteins Lhca2*1 and Lhcb4*2 [36]. Down-regulation of photosynthesis genes is accompanied by a sudden suppression of photosynthesis at low temperatures [37]. However this might be a transient effect, since leaves that developed at low temperatures are reported to regain full photosynthetic activity [38]. Although a reduction in antenna size would not necessarily impact parameters Ф_PSII_ and qP, it is not self-evident why low temperatures should increase effective quantum yield. Savitch & co-workers [38] reported that low temperatures could increase photosynthetic capacity in species like e.g. wheat that maintain a high need for assimilates because of active growth at low temperatures. In the cold tolerant tomato species *Lycopersicon peruvianum*, a rise in qP during cold exposure was hypothesized to result from increased capacity of the Calvin-Benson cycle [39]. In this context it should be noted that temperature effects on light absorption and photosynthesis are often not easily separable from light effects, because in most studies light intensity is reduced at low temperature to avoid damage to the photosystems in the cold. At lower light intensity, qN is reduced and qP rises, and in turn also Ф_PSII_ is increased as long as no damage of the photosystems occurs. This effect may superimpose the temperature effect on light harvesting, and although the cold sensitive accession Cvi could be discriminated from all intermediate and tolerant accessions based on the lack of increase of Ф_PSII_ during acclimation, this parameter does not seem to be sufficient for screening cold tolerance in acclimated *Arabidopsis* plants.

### Combinatorial imaging can discriminate cold acclimation induced cold tolerant plants

We have earlier reported that combinatorial imaging of ChlF transients combined with classifier and feature selection methods was able to discriminate detached leaves from cold sensitive, tolerant and intermediate accessions of Arabidopsis [10]. The main outcome of the current study is that this method can also be applied to whole plants, i.e. leaves to be compared need not be present on the same images, and thus this method is suitable for large scale screening of plant cold tolerance.

Combinatorial imaging combines sets of highly performing images from sequences of time-resolved ChlF images that provide strong discrimination capacity [10, 29, 30]. High performing images extract information from several thousands of pixels from hundreds of measured images (218 images per data for this experiment, and each image with resolution 512*512 pixels). This method does not trace underlying physiological phenomena rather its algorithms select image sets having optimal contrast between sensitive and tolerant features that ultimately allow discrimination on the basis of their cold tolerance levels [30]. Therefore, parameters identified for the classifiers are very likely species and treatment specific, and training of the classifiers needs to be performed for each application of the method. While this might appear disadvantageous, it also offers great flexibility, because successful discrimination is not dependent on any specific physiological process but can exploit every feature that allows discrimination [10, 29, 30, 40, 41]. In the current study, older leaves performed better than developing ones, probably because the higher fluorescence intensity provided a better signal-to-noise ratio. However, this does not implicate that the method is not suited to the screening of plant seedlings, because it analyzes relative fluorescence intensities distributed among the image pixels and for each sample type it is different [29]. Therefore, combinatorial imaging out-performs existing methods that depend on the application of freeze-thaw treatments.

The combinatorial imaging method could discriminate sensitive and tolerant accessions in the cold acclimated state but it failed to make any difference between cold sensitive and intermediate accessions. Because training was done for STT plants, this failure could result from differential responses of the photosynthetic apparatus to low above-zero and sub-zero temperatures as outlined above, or it could relate to insufficient resolution, which might be overcome by a comparison of larger sets of images. Since this would clearly result in higher computational load, a case specific decision for higher resolution vs. shorter analysis time might be necessary.

Even if only a two-class discrimination can be achieved with the combinatorial imaging method, it would be very useful for large scale screening of cold tolerance e.g. in recombinant inbred line populations or other plant sets consisting of large numbers of individuals representing different genotypes. The method is thus well suited for quantitative trait loci (QTL) mapping or mutant screenings to investigate genetic determinants of cold tolerance that may be used for plant breeding.

## Conclusions

We have demonstrated that chlorophyll fluorescence emission from whole plant rosettes of *Arabidopsis thaliana* integrates information that can be used to discriminate cold sensitive and tolerant plants in the cold acclimated state when analyzed by the advanced statistics based combinatorial imaging approach [10, 29, 30]. This reveals the power of combinatorial imaging for identifying features of cold tolerant and sensitive accessions at cold acclimation state (Figure 5) where well known physiological parameters of ChlF emission (Figures 2 and 3) failed to provide any clue of discrimination. Moreover, capturing ChlF transients of whole plant rosettes following mild sub-zero temperature treatments (STT, -4°C for ~8 h in dark) is also very useful, because classical ChlF transients and the extracted parameters such as F_V_/F_M_ and R_FD_ can categorize cold sensitive, intermediate and tolerant accessions following STT. In addition, classical ChlF analysis, in contrast to combinatorial imaging methods, can yield physiologically relevant information that could be directly exploited for breeding efforts. The screening of whole plants cold tolerance *via* fully non-invasive means following cold acclimation, i.e. without any sub-zero temperature treatments, could be highly useful in high-throughput screening of cold tolerance where it is superior to data measured from single leaf or leaf discs by alternative methods such as metabolomics [7] or electrolyte leakage [5].

## Methods

### Plant material and growth conditions

Six plantlets of each of the nine accessions of *Arabidopsis thaliana* [5] were grown inside a cooling chamber in pots of 0.06 m for six weeks at day/night temperatures 20°C/18°C, light 90 μmol (photons) m^-2^ s^-1^ and a relative humidity of 70%. The accessions used in this study were: Cvi (Cap Verde Islands), Can (Canary Islands), C24 (genetically related to Co, Portugal), Co (Coimbra, Portugal), Col-0 (Columbia-0, genetically related to Gü, Germany), Nd (Niederzenz, Germany), Ler (Landsberg *erecta,* Poland), Rsch (Rschew, Russia), and Te (Tenela, Finland). The experiments were executed in three independent replica with three independent sets of plants used to measure ChlF in the non acclimated (NAC, six weeks grown plants), cold acclimated (AC, NAC plants acclimated at 4°C for another 2 weeks), and sub-zero temperature treated (STT, AC plants treated with mild sub-zero temperature of -4°C for 8 hours in dark) state.

### Chlorophyll fluorescence measurement

Individual plants were used for ChlF measurements at room temperature using Handy FluorCam [http://www.psi.cz; [23]. A short protocol of ~202 seconds modified according to Mishra et al. [10] was used for capturing time-resolved ChlF from plant rosettes. This protocol starts with the measurement of basal fluorescence (F_O_) and maximum fluorescence (F_M_) using measuring and saturating flashes. After a short dark period of ~20 seconds, actinic light of 40 μmol (photons) m^-2^ s^-1^ was applied to measure the fluorescence transients. Two strong flashes of saturating light were overlaid with actinic light to investigate activation of non-photochemical quenching followed by a third saturating flash 18 s after switching off the actinic light to see the relaxation of non-photochemical quenching mechanisms. Three replicate recordings were taken for NAC, AC and STT state, respectively. The images of the measured ChlF transients were averaged across the whole rosettes for quantitative evaluation of the fluorescence parameters or plotting of ChlF transients.

### Combinatorial imaging using statistical classifier and feature selection method

The method of combinatorial imaging was applied to identify the discriminant between the accessions without using the whole data set consisting of 218 images in a time series. Each time series contains some repetitive images as well as images with low contrast, which were sorted out to reduce the size of the data sets. Application of statistical techniques of classifiers was chosen to avoid biasness. We randomly classified the data for Te and Co as training and testing sets. Using the training set, features discriminating the tolerant and sensitive accession were identified, and these features were next applied using the testing set for discriminating cold tolerant and sensitive accessions. Using this method we calculated the performance of several classifiers: LDC, QDC, *k*-NNC, NNC and NMC, and chose the best performing classifier for further analysis. The performance of each of the investigated classifiers was quantified by a number between 0–1: value‘0’ means random classification (1/2 of classifications into 2 equally represented classes correct, and another 1/2 is incorrect) and value ‘1’ meaning that the classifier was 100% successful [for details see [24]. In this experiments performance of QDC was 81% and computational time was less than 6 seconds. Therefore, QDC classifier was applied with sequential forward floating selection (SFFS) to reduce the number of images for effective classification [42]. The reduction is based on finding an image sub-set containing the most useful information for the visualization of highly contrasting features of cold tolerant vs. sensitive accessions and arranging the images in a form of descending order of their performance efficiency. The combinatorial images are developed by the combination of the three high performing images (x,y,z) multiplied with their coefficient (a,b,c) that are obtained by *linear discriminant analysis* (LDA): Combinatorial imaging (C) = (±a)*I_x_ + (±b)*I_y_ + (±c)*I_z_.

### Tool for the data analysis

The Matlab software package, version 6.5, with Pattern Reorganization toolbox (PRTools) was used for statistical analysis.

## Abbreviations

ChlF: Chlorophyll *a* fluorescence
NAC: Non-acclimated
AC: Cold acclimated
ST(T): Sub-zero temperature (treated)
PSII: photosystem II
PSI: Photosystem I
F_V_/F_M_: Maximum quantum yield of PSII photochemistry, where F_V_ is variable fluorescence (F_V_ = F_M_-F_O_), F_O_ is minimal fluorescence emission of a dark adapted plant with primary quinone acceptor (Q_A_), oxidized and non-photochemical quenching inactive, and F_M_ is maximum fluorescence emission of a dark adapted plant exposed to a short pulse of a strong light leading to a transient reduction of Q_A_
R_FD_ = (F_P_-F_S_)/F_S_: Fluorescence decrease ratio, where F_P_ is a fluorescence peak at the beginning of the transient with actinic light, and F_S_ is steady state fluorescence
QDC: Quadratic discriminant classifier
SFFS: Sequential forward floating selection
EL: Electrolyte leakage
LT_50_: The half of maximal lethal temperature
EL: Electrolyte leakage
Ф_PSII_: Effective quantum efficiency of PSII photochemistry
qP: Photochemical quenching
qN: Non-photochemical quenching
*k*-NNC: *k*- nearest neighbor classifier
NNC: Nearest neighbor classifier
NMC: Nearest mean classifier
LDA: Linear discriminant analysis.
